# Integrated transcriptome and miRNA analysis uncovers molecular regulators of aerial stem-to-rhizome transition in the medical herb *Gynostemma pentaphyllum*

**DOI:** 10.1186/s12864-019-6250-8

**Published:** 2019-11-15

**Authors:** Qi Yang, Shibiao Liu, Xiaoning Han, Jingyi Ma, Wenhong Deng, Xiaodong Wang, Huihong Guo, Xinli Xia

**Affiliations:** 1 Beijing Advanced Innovation Center for Tree Breeding by Molecular Design, National Engineering Laboratory for Tree Breeding, College of Biological Sciences and Biotechnology, Beijing Forestry University, No. 35, Tsing Hua East Road, Haidian District, Beijing, 100083 China; 2 College of Biology and Environmental Sciences, Jishou University, Jishou, 416000 China; 3 Analytical and Testing Center, Beijing Forestry University, Beijing, 100083 China; 4 Centre for Imaging & Systems Biology, College of Life and Environmental Sciences, Minzu University of China, Beijing, China

**Keywords:** *Gynostemma pentaphyllum*, Aerial stem-to-rhizome transition, Transcriptome, miRNAs, Integrated analysis

## Abstract

**Background:**

*Gynostemma pentaphyllum* is an important perennial medicinal herb belonging to the family Cucurbitaceae. Aerial stem-to-rhizome transition before entering the winter is an adaptive regenerative strategy in *G. pentaphyllum* that enables it to survive during winter. However, the molecular regulation of aerial stem-to-rhizome transition is unknown in plants. Here, integrated transcriptome and miRNA analysis was conducted to investigate the regulatory network of stem-to-rhizome transition.

**Results:**

Nine transcriptome libraries prepared from stem/rhizome samples collected at three stages of developmental stem-to-rhizome transition were sequenced and a total of 5428 differentially expressed genes (DEGs) were identified. DEGs associated with gravitropism, cell wall biosynthesis, photoperiod, hormone signaling, and carbohydrate metabolism were found to regulate stem-to-rhizome transition. Nine small RNA libraries were parallelly sequenced, and seven significantly differentially expressed miRNAs (DEMs) were identified, including four known and three novel miRNAs. The seven DEMs targeted 123 mRNAs, and six pairs of miRNA-target showed significantly opposite expression trends. The GpmiR166b-*GpECH2* module involved in stem-to-rhizome transition probably promotes cell expansion by IBA-to-IAA conversion, and the GpmiR166e-*GpSGT*-*like* module probably protects IAA from degradation, thereby promoting rhizome formation. GpmiR156a was found to be involved in stem-to-rhizome transition by inhibiting the expression of *GpSPL13A*/*GpSPL6*, which are believed to negatively regulate vegetative phase transition. GpmiR156a and a novel miRNA Co.47071 co-repressed the expression of growth inhibitor *GpRAV*-*like* during stem-to-rhizome transition. These miRNAs and their targets were first reported to be involved in the formation of rhizomes. In this study, the expression patterns of DEGs, DEMs and their targets were further validated by quantitative real-time PCR, supporting the reliability of sequencing data.

**Conclusions:**

Our study revealed a comprehensive molecular network regulating the transition of aerial stem to rhizome in *G. pentaphyllum.* These results broaden our understanding of developmental phase transitions in plants.

## Background

*Gynostemma pentaphyllum* (Thunb.) Makino, belonging to the genus *Gynostemma* in the family Cucurbitaceae, is a perennial herb widely distributed in Asian countries [1]. *G. pentaphyllum* contains important medicinal components, called gypenosides, which are reportedly effective in the treatment of various illnesses, such as inflammation, cardiovascular diseases, and cancer [2–4]. This herb is widely used as tea or functional food [5], and has thus received substantial attention in recent years.

*G. pentaphyllum* is a dioecious, herbaceous vine with a female-to-male ratio of 1:20, which is not conducive to seed production [6]. Moreover, its seeds contain germination inhibitors and exhibit deep dormancy at maturity, and thus, it propagates mainly vegetatively under natural conditions [7]. The aboveground part of the vine lives only 1 year and dies in winter under natural conditions. Interestingly, before entering the winter, the subapical regions of some aerial stems swell and then drill into the soil to form rhizomes that produce new plants in the next year [6]. This vegetative regeneration is an adaptation of *G. pentaphyllum* to the natural environment to maintain its population. Aerial stem-to-rhizome transition implies not only morphological changes, but also functional changes in processes ranging from transport and support to storage and reproduction. This developmental phase transition is an interesting research topic in the field of developmental biology.

Accumulating evidence shows that plant developmental phase transitions involve the regulation of a large numbers of genes [8–11]. For example, transcriptome analysis revealed that genes related to the photoperiod pathway, starch biosynthesis, and hormone signaling are involved in stolon-to-rhizome transition in lotus [9]. miRNAs have also been confirmed to be involved in plant developmental phase transitions [12, 13]. miRNAs are single-stranded small noncoding RNAs of 20–24 nt in length that repress the expression of target genes by transcript cleavage and/or translation inhibition [14]. The identification of miRNA targets is critical for functional investigation of miRNAs. For example, miR156 and miR172 targets *SQUAMOSA PROMOTER BINDING PROTEIN-LIKE* (*SPL*) and *APETALA2* (*AP2*) regulate juvenile-to-adult and adult-to-reproduction transitions, respectively, in *Arabidopsis* [8]. miR156 is also involved in the regulation of tuberization in potato, and miR156 abundance increases in stolons under tuber-inductive conditions [15]. The miR159-*MYB33* module controls the transition from the vegetative to the reproductive phase, and enhanced miR159 expression delayed flowering time in *Arabidopsis* [16]. miR166 affects root development by targeting several homeodomain-leucine zipper (*HD-ZIP*) genes in *Medicago truncatula* [17], whereas the miR166-*PHABULOSA* module participates in the embryogenic transition of somatic cells in *Arabidopsis* [18]. More recently, novel miRNAs involved in potato tuber formation have been identified [13]. To date, little is known about whether and which miRNAs participate in aerial stem-to-rhizome transition in plants. Except for miRNAs and their targets, it is also unknown which other genes are involved in aerial stem-to-rhizome transition.

In this study, we conducted integrated transcriptome and miRNA analyses to investigate the molecular mechanism underlying aerial stem-to-rhizome transition in *G. pentaphyllum*. We expected our findings to broaden our understanding of developmental transitions in plants.

## Results

### Morphological and histological traits of aerial stem-to-rhizome transition in *G. pentaphyllum*

**Fig. 1:**
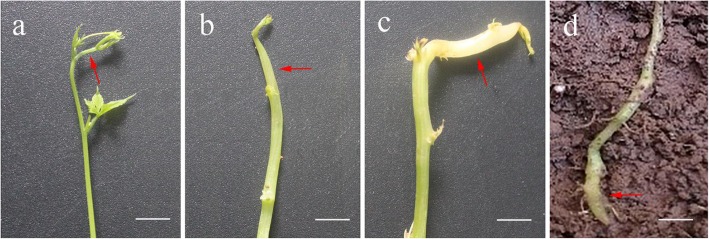
Morphological traits at different stages in aerial stem-to-rhizome transition in *Gynostemma pentaphyllum*. **a** Aerial stem (stage 1). **b** Aboveground moderately swelling stem (stage 2). **c**, **d** Underground newly formed rhizome (stage 3). Red arrows indicate sampling position. Bar = 10 mm

As shown in Fig. 1, aerial stem, aboveground moderately swelling stem, and underground newly formed rhizome were selected as representative stages of developmental aerial stem-to-rhizome transition in *G*. *pentaphyllum* and were named stage 1, stage 2, and stage 3, respectively. In the process of stem-to-rhizome transition, the subapical regions of aerial stems swelled and expanded away from the tip, and then grew down into the soil. As swelling intensified, the stem diameter increased by about 1, 3 and 5 mm at the three developmental stages, respectively. Correspondingly, the stem color changed gradually from green to pale green, and finally to white (Fig. 1a-c). Rhizome, as a modified subterranean stem, exhibited anatomical characteristics similar to those of aerial stem (Additional file 1: Figure S1). This result is consistent with a recent report on *Oryza longistaminata* [19]. Stems at transition stages 1, 2, and 3 were all composed of epidermis, cortex, vascular bundles arranged along the stem circumference, and pith from outside to inside (Additional file 1: Figure S1). It is noteworthy that there is a circle of perivascular fibers composed of several layers of cells outside the vascular bundles (Additional file 1: Figure S1). Histochemical observation revealed that only a small amount of starch grains accumulated in stage 1 and stage 2 stems, whereas more and larger starch grains were present in stems at stage 3 (Additional file 2: Figure S2). The starch grains mainly accumulated in the innermost layer of the cortex, termed the starch sheath, and the pith (Additional file 2: Figure S2). In stage 3, starch grains accumulated even in the phloem parenchyma cells of the vascular bundles (Additional file 2: Figure S2).

### Transcriptome analysis of aerial stem-to-rhizome transition in *G. pentaphyllum*

#### RNA-Seq and de novo assembly

**Fig. 2:**
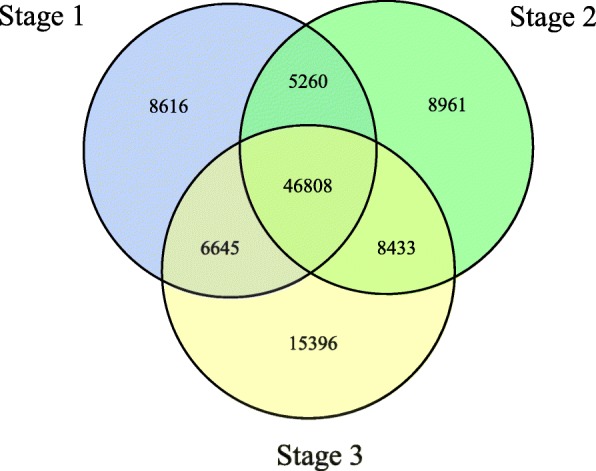
Venn diagram showing the numbers of genes expressed in each of the three developmental stages

To explore the molecular basis of aerial stem-to-rhizome transition, RNA-Sequencing (RNA-Seq) was conducted to generate transcriptome profiles. Nine RNA libraries derived from the above-mentioned three developmental stages of aerial stem-to-rhizome transition were sequenced on an Illumina HiSeq X Ten platform. In total, 352,070,555 cleaned reads were generated (Table 1). *De-novo* assembly of the cleaned reads yielded 207,635 transcripts, which were further assembled into 100,119 unigenes with an N50 length of 1336 bp (Additional file 9: Table S1). E90N50 value was 2658 bp, which represents the N50 of 90% of the total normalized expressed transcripts (Additional file 3: Figure S3b). Bench-marking universal single-copy orthologs (BUSCO) analysis showed a completeness score of 66.4%, a fragmented score of 23.4 and 10.2% as missing BUSCOs (Additional file 10: Table S2). The length distribution of the unigenes is shown in Additional file 3: Figure S3a, and Fig. 2 shows the genes that are similarly and distinctly regulated among the three stages. In total, 46,808 genes were expressed in all three stages, whereas 8616, 8961, and 15,396 genes were uniquely expressed in stage 1, stage 2, and stage 3, respectively (Fig. 2). These stage-specific expressed genes that were primarily assigned to carbon metabolism, amino acid biosynthesis, and ribosomes at each developmental stage, indicating that they exhibited different temporal and spatial expression patterns during the aerial stem-to-rhizome transition of *G. pentaphyllum*. Because of the lack of a reference genome sequence, the cleaned reads were mapped onto the assembled transcriptome; 81.16% of cleaned reads were aligned (Table 1). Principal component analysis (PCA) revealed that three samples from the same stage were clustered together and nine samples from three stages were clearly assigned to three groups as stage 1, stage 2 and stage 3 (Additional file 3: Figure S3c).

**Table 1:** Summary of RNA-Seq data and mapping statistics

Library	Cleaned Reads	GC Content (%)	Q30 (%)	Mapped Reads	Ratio
Stage 1–1	42,774,586	42.59%	93.65%	34,967,471	81.75%
Stage 1–2	41,570,187	42.73%	93.78%	34,017,316	81.83%
Stage 1–3	34,789,353	42.68%	93.20%	28,593,464	82.19%
Stage 2–1	40,858,136	42.61%	93.22%	33,497,321	81.98%
Stage 2–2	35,399,865	42.85%	93.03%	29,207,997	82.51%
Stage 2–3	40,375,395	42.66%	92.97%	33,093,255	81.96%
Stage 3–1	43,859,253	43.07%	92.61%	35,113,752	80.06%
Stage 3–2	35,807,302	42.66%	93.23%	29,097,263	81.26%
Stage 3–3	36,636,478	42.68%	93.16%	29,739,162	81.17%
Total	352,070,555	–	–	287,327,001	81.16%

Q30 (%): bases with a quality value > 30; Ratio: the ratio of mapped reads to cleaned reads

#### Identification and functional classification of differentially expressed genes

To identify differentially expressed genes (DEGs), pairwise comparisons were conducted among the three stages of *G. pentaphyllum* aerial stem-to-rhizome transition. In total, 5428 DEGs were filtered out based on FDR < 0.01 and |log_2_ fold change| ≥1 in each pairwise comparison (Additional file 11: Table S3); 1683 and 792 genes were significantly up- and downregulated, respectively, in the transition from stage 1 to stage 2; and 906 and 763 genes were significantly up- and downregulated, respectively, in that from stage 2 to stage 3 (Additional file 3: Figure S3c). In the transition from stage 1 to stage 3, 2552 and 2075 genes were significantly up- and downregulated, respectively (Additional file 3: Figure S3d).

Five thousand four hundred twenty-eight DEGs were annotated using blastx and the functions of these DEGs were investigated by Gene Ontology (GO) and Kyoto Encyclopedia of Genes and Genomes (KEGG) analyses (Additional file 4: Figure S4 and Additional file 5: Figure S5). Stage 1-to-stage 2 DEGs were predominantly involved in hormone signal transduction, phenylpropanoid biosynthesis, carbon metabolism, ribosome, photosynthesis, and starch and sucrose metabolism (Additional file 5: Figure S5a). Among them, upregulated DEGs were mainly assigned to hormone signal transduction, phenylpropanoid biosynthesis, and starch and sucrose metabolism, whereas downregulated DEGs were mainly involved in photosynthesis, ribosome, and carbon metabolism (Additional file 5: Figure S5b-c). Similar findings were obtained for stage 2-to-stage 3 DEGs (Additional file 5: Figure S54e-f).

#### DEGs related to the aerial stem-to-rhizome transition

Aerial stem-to-rhizome transition involves a conversion from negative to positive gravitropism. Genes encoding indole-3-pyruvate monooxygenase (YUCCA), LAZY1, and actin-related protein 2/3 (ARP2/3) complex reportedly are involved in gravitropism [20]. Putative homologs of these genes were identified in *G. pentaphyllum* (Table 2). Among them, two *GpYUCCA* and *GpLAZY1* were respectively well clustered with their homologs whose functions have been reported to be associated with gravitropism (Additional file 8: Figure S8). The expressions of these genes was significantly upregulated during the transition from stage 1 to stage 3 (Table 2).

**Table 2:** Annotation of gravitropism-related DEGs identified in pairwise comparisons of stages in developmental aerial stem-to-rhizome transition of *Gynostemma pentaphyllum*

Gene ID	Gene name	log_2_ Fold Change			Annotation
		Stage 1 vs Stage 2	Stage 2 vs Stage 3	Stage 1 vs Stage 3	
c47850.graph_c0	*GpYUCCA*-*a*	0.73	0.44	1.22^a^	Indole-3-pyruvate monooxygenase
c48164.graph_c0	*GpYUCCA*-*b*	1.13^a^	0.71	2.02^a^	Indole-3-pyruvate monooxygenase
c51192.graph_c0	*GpLAZY1*	1.34^a^	0.11	1.43^a^	LAZY 1
c54974.graph_c0	*GpARP2/3*	1.48^a^	0.58	2.09^a^	Actin related protein 2/3 complex

^a^DEGs with FDR < 0.01, |log_2_ fold change| ≥1

**Fig. 3:**
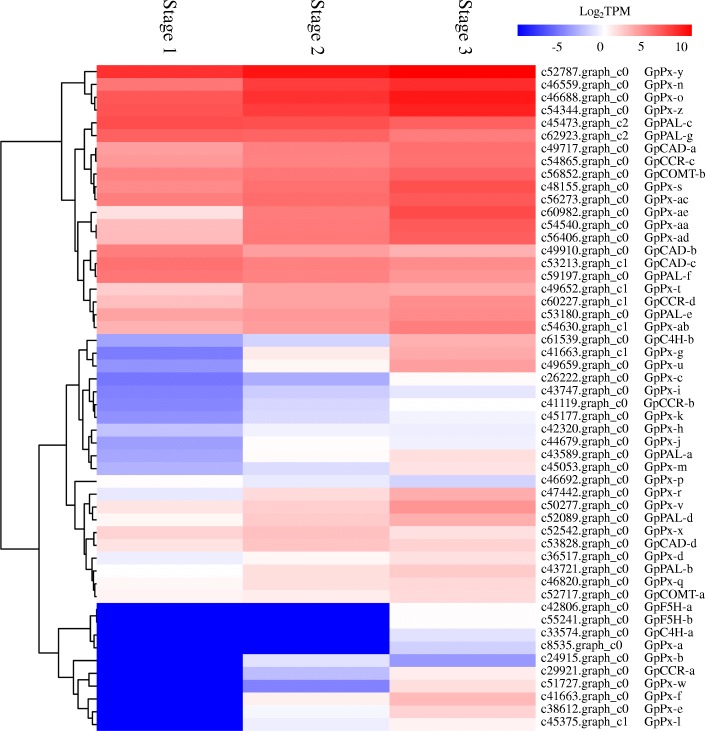
Heatmap of putative DEGs involved in phenylpropanoid biosynthesis in aerial stem-to-rhizome transition of *Gynostemma pentaphyllum*

Phenylpropanoid biosynthesis is involved in rhizome formation [21]. In this study, a large number of DEGs was assigned to the phenylpropanoid pathway, including genes encoding phenylalanine ammonia-lyase (PAL), trans-cinnamate 4-monooxygenase (C4H), caffeic acid 3-O-methyltransferase (COMT), ferulate-5-hydroxylase (F5H), cinnamoyl-CoA reductase (CCR), cinnamyl-alcohol dehydrogenase (CAD), and peroxidase (Px) (Fig. 3). Most of the putative genes encoding these enzymes were significantly upregulated during aerial stem-to-rhizome transition of *G. pentaphyllum*. Among them, some genes were upregulated at stage 2, whereas others were upregulated at stage 3 when compared with stage 1 (Fig. 3).

Rhizome formation is also controlled by distinct photoperiod-related genes [22]. Some genes encoding phytochrome A (PHYA), CONSTANS-like (COL) protein, cyclic dof factor (CDF), and flavin-binding kelch repeat F-box protein 1 (FKF1) in the photoperiod pathway were identified (Table 3). Among them, *GpCOLs* and *GpCDF* were respectively well clustered with their homologs whose functions have been reported to be involved in photoperiod (Additional file 8: Figure S8). Genes encoding PHYA, FKF1, and two out seven of genes encoding COL were significantly upregulated in stage 3 compared to stage 1, whereas genes encoding CDF and five out seven of genes encoding COL were significantly downregulated during aerial stem-to-rhizome transition.

**Table 3:** Annotation of photoperiod pathway-related DEGs identified in pairwise comparisons of stages in developmental aerial stem-to-rhizome transition of *Gynostemma pentaphyllum*

Gene ID	Gene name	log_2_ Fold Change			Annotation
		Stage 1 vs Stage 2	Stage 2 vs Stage 3	Stage 1 vs Stage 3	
c62585.graph_c0	*GpPHYA*	0.65	0.85	1.48^a^	Phytochrome A
c47576.graph_c0	*GpCOL*-*a*	−0.66	− 0.90	−1.58^a^	CONSTANS-like
c47991.graph_c0	*GpCOL*-*b*	−0.02	−1.08^a^	−1.18^a^	CONSTANS-like
c48345.graph_c0	*GpCOL*-*c*	1.00	0.50	1.64^a^	CONSTANS-like
c49299.graph_c0	*GpCOL*-*d*	−1.05^a^	−0.18	−1.26^a^	CONSTANS-like
c50325.graph_c0	*GpCOL*-*e*	1.17^a^	2.03^a^	3.20^a^	CONSTANS-like
c52252.graph_c0	*GpCOL*-*f*	−0.47	−1.50^a^	−2.12^a^	CONSTANS-like
c53693.graph_c0	*GpCOL*-*g*	−0.63	−1.87^a^	− 2.62^a^	CONSTANS-like
c62923.graph_c1	*GpCDF*	−1.68^a^	−0.18	−1.95^a^	Cyclic dof factor
c51745.graph_c0	*GpFKF1*	−0.15	1.65^a^	1.55^a^	Flavin-binding kelch repeat F-box protein 1

^a^DEGs with FDR < 0.01, |log_2_ fold change| ≥1

**Fig. 4:**
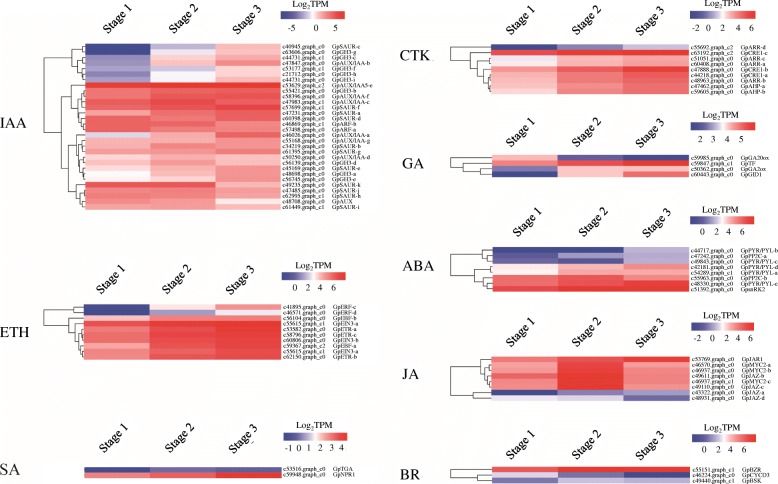
Heatmap of hormone signaling-related DEGs putatively involved in aerial stem-to-rhizome transition of *Gynostemma pentaphyllum*

Plant hormones play crucial roles in rhizome formation [10]. Seventy-four genes associated with the biosynthesis, metabolism, and signaling of plant hormones, including gibberellin acid (GA), abscisic acid (ABA), ethylene (ETH), cytokinin (CTK), auxin (IAA), brassinosteroid (BR), jasmonic acid (JA), and salicylic acid (SA), were identified (Fig. 4). It is noteworthy that 30 genes were assigned to the IAA signaling pathway, and their expression was generally significantly upregulated. Genes related to the ETH, CTK, and SA pathways were significantly upregulated in stage 3 compared to stage 1. Most genes involved in the biosynthesis, metabolism, and signaling of GA (3 out of 4), ABA (7 out of 8), IAA (21 out of 30), BR (2 out of 3), and JA (7 out of 8) were also significantly upregulated during aerial stem-to-rhizome transition, except for several downregulated genes, including *GA20ox* (Fig. 4).

Carbohydrate metabolism-related starch biosynthesis is strongly involved in the development and function of storage organs, including rhizomes, corms, tubers, and bulbs [23]. Several putative genes encoding sucrose synthase (SUS), granule-bound starch synthase (GBSS), cellulose synthase (CESA), and SNF1-related protein kinase regulatory subunit gamma-1 (KING1) were found to be significantly upregulated during aerial stem-to-rhizome transition of *G. pentaphyllum* (Table 4). These genes have been suggested to be closely related to carbohydrate metabolism [23, 24].

**Table 4:** Annotation of carbohydrate metabolism-related DEGs identified in pairwise comparisons of stages in developmental aerial stem-to-rhizome transition of *Gynostemma pentaphyllum*

Gene ID	Gene name	log_2_ Fold Change			Annotation
		Stage 1 vs Stage 2	Stage 2 vs Stage 3	Stage 1 vs Stage 3	
c57893.graph_c0	*GpSUS*-*a*	0.44	4.17^a^	4.62^a^	Sucrose synthase
c58010.graph_c0	*GpSUS*-*b*	0.60	0.59	1.18^a^	Sucrose synthase
c63160.graph_c0	*GpGBSS*	0.41	0.73	1.12^a^	Granule-bound starch synthase
c51009.graph_c0	*KING1*	1.95^a^	2.25^a^	4.23^a^	SNF1-related protein kinase regulatory subunit gamma-1
c55307.graph_c0	*GpCESA*-*a*	3.68^a^	0.67	4.34^a^	Cellulose synthase A
c59421.graph_c0	*GpCESA*-*b*	3.47^a^	0.63	4.08^a^	Cellulose synthase A
c58320.graph_c0	*GpCESA*-*c*	1.51^a^	0.42	1.92^a^	Cellulose synthase A

^a^DEGs with FDR <0.01, |log_2_ fold change| ≥1

### miRNAs and miRNA targets involved in aerial stem-to-rhizome transition in *G. pentaphyllum*

#### Sequencing of small RNAs and identification of miRNAs

Nine small RNA libraries from three stages in developmental aerial stem-to-rhizome transition of *G. pentaphyllum* were generated and sequenced. In total, 281,846,013 cleaned reads were obtained (Table 5). Among them, 204,459,222 cleaned reads, accounting for 72.54% of the total cleaned reads, could be mapped to known small RNA databases (Table 5). The mapped reads were categorized into seven classes, including miRNA (4.91%), ribosomal RNA (rRNA, 64.57%), transfer RNA (tRNA, 2.78%), small nucleolar RNA (snoRNA, 0.10%), repeats (0.18%), and unannotated reads (27.46%) (Additional file 12: Table S4). In total, 90 known miRNAs were identified by mapping the cleaned reads to known plant miRNA databases (Additional file 13: Table S5). The remaining unmapped reads were used to predict novel miRNAs; 158 novel miRNAs were identified (Additional file 13: Table S5). These miRNAs were mainly 20–24 nt in length, and the most abundant miRNAs were 21 nt in length (Additional file 6: Figure S6).

**Table 5:** Statistics of small RNA-Seq data and mapping

Library	Cleaned Reads	Q30 (%)	Mapped Reads	Ratio
Stage 1–1	25,419,864	99.52	17,280,653	67.98%
Stage 1–2	27,725,314	99.51	20,788,388	74.98%
Stage 1–3	32,083,772	99.47	24,038,271	74.92%
Stage 2–1	29,680,316	98.97	22,967,955	77.38%
Stage 2–2	27,980,952	98.73	20,590,376	73.59%
Stage 2–3	27,524,015	98.92	19,633,005	71.33%
Stage 3–1	28,148,315	99.47	21,457,794	76.23%
Stage 3–2	58,751,076	98.69	39,615,210	67.43%
Stage 3–3	24,532,389	99.50	18,087,570	73.73%
Total	281,846,013	–	204,459,222	72.54%

Q30 (%): bases with a quality value > 30; Ratio: the ratio of mapped reads to cleaned reads

### Identification of differentially expressed miRNAs

To identify differentially expressed miRNAs (DEMs), pairwise comparisons were performed among the three transition stages based on the criteria of FDR < 0.01 and |log_2_ fold change| ≥1. Four known and three novel miRNAs were significantly differentially expressed during aerial stem-to-rhizome transition (Table 6). In the transition from stage 1 to stage 2, GpmiR156a, GpmiR159, and Co.47071 were significantly upregulated, whereas Co.25160 and Co.59333 were significantly downregulated; between stages 2 and 3, GpmiR156a and Co.47071 were significantly upregulated, whereas Co.25160 was significantly downregulated. During the transition from stage 1 to stage 3, GpmiR156a, GpmiR159, and Co.47071 were significantly upregulated, whereas GpmiR166b-5p, GpmiR166e-3p, Co.25160, and Co.59333 were significantly downregulated.

**Table 6:** Differentially expressed miRNAs (DEMs) from pairwise comparisons among stages of developmental aerial stem-to-rhizome transition in *Gynostemma pentaphyllum*

miRNA ID	log_2_ Fold Change		
	Stage 1 vs Stage 2	Stage 2 vs Stage 3	Stage 1 vs Stage 3
GpmiR166b-5p	−0.45	−0.75	−1.19^a^
GpmiR166e-3p	−0.34	−0.89	−1.23^a^
GpmiR156a	2.30^a^	1.42^a^	3.24^a^
GpmiR159	1.02^a^	0.76	1.79^a^
Co.25160	−2.82^a^	−4.78^a^	−7.54^a^
Co.47071	3.30^a^	1.55^a^	4.87^a^
Co.59333	−1.17^a^	0.15	−1.02

^a^DEMs with FDR < 0.01, |log_2_ fold change| ≥1

#### Identification and functional annotation of mRNA targets of differentially expressed miRNAs

**Fig. 5:**
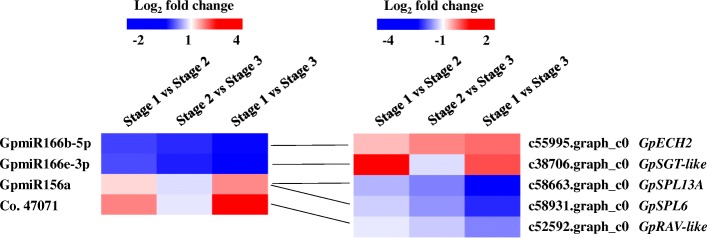
Heatmap of differentially expressed miRNAs with their target genes during aerial stem-to-rhizome transition of *Gynostemma pentaphyllum*

In total, 123 putative targets for GpmiR166b-5p, GpmiR166e-3p, GpmiR156a, GpmiR159, Co.47071, and Co.59333 were identified, whereas Co.25160 had no predicted target genes (Additional file 14: Table S6). Six miRNA-target pairs exhibiting contrasting expression trends were identified (|log_2_ fold change| ≥1; FDR *<* 0.01) (Fig. 5). GpmiR166b-5p and GpmiR166e-3p were significantly downregulated during aerial stem-to-rhizome transition, and their target genes, which encode enoyl-CoA hydratase 2 (ECH2) and scopoletin glucosyltransferase (SGT)-like, respectively, were significantly upregulated (Fig. 5). In contrast, GpmiR156a and miRNA Co.47071 were significantly upregulated during the transition and their targets were significantly downregulated (Fig. 5). Among them, GpmiR156a targeted two *SPL* transcription factor genes (*GpSPL6/GpSPL13A*) and a gene encoding related to ABI3/VP1 (RAV)-like factor. Like GpmiR156a, miRNA Co.47071 also targeted the *GpRAV*-*like* gene (Fig. 5).

#### Validation of DEGs, DEMs, and their targets

To validate the DEGs, DEMs, and their targets identified by Illumina sequencing, 32 representative genes, five DEMs, and six miRNA-target pairs were investigated by quantitative real-time PCR (qRT-PCR). The qRT-PCR results were consistent with the sequencing data, supporting the reliability of sequencing data (Additional file 7: Figure S7).

## Discussion

### Transcriptomic analysis reveals the important roles of DEGs involved in *G. pentaphyllum* aerial stem-to-rhizome transition

RNA-Seq is a powerful and efficient means to discover putative functional genes involved in diverse biological processes, especially for plant species without a reference genome [10]. Using this tool, we found 5428 genes to be differentially expressed during stem-to-rhizome transition in *G. pentaphyllum*. Among them, DEGs were mostly related to gravitropism, phenylpropanoid biosynthesis, photoperiod, hormone synthesis and signal transduction, and carbohydrate metabolism.

Gravitropism is vital for shaping directional growth of plants in response to gravity [25]. Shoots grow upward (negative gravitropism), whereas roots grow downward (positive gravitropism) due to a gravitropic response, which results in differential growth between upper and lower sides of these organs [26]. Differential growth is thought to be controlled by polar auxin transport and asymmetric auxin distribution in different parts of graviresponding organs [27]. In this study, several homologs of known gravitropism-related genes, including *GpYUCCA*-*a*, *GpYUCCA*-*b, GpLAZY1*, and *GpARP2/3*, were significantly upregulated (Table 2). *YUCCA* genes encode flavin monooxygenases that catalyze a key step in the conversion of tryptophan into IAA [28]. In *Arabidopsis*, mutation of five *YUCCA* genes led to IAA deficiency and abnormal gravitropic responses of roots [29]. *LAZY1* and *ARP2/3* also play essential roles in shoot and root gravitropism by affecting polar auxin transport and asymmetric auxin distribution [20, 30, 31]. Thus, it is suggested that these gravitropism-related DEGs cooperatively control the gravitropic response during aerial stem-to-rhizome transition, probably by promoting auxin biosynthesis and altering auxin polar transport and distribution, thereby enabling the rhizome to acquire a positive gravitropism phenotype and to thus grow into the soil.

The phenylpropanoid pathway generates lignin precursors, which are transported into the cell walls for polymerization into lignin [32]. Lignin is mainly present in sclerenchymatous cells, such as vessel and fiber, whose lignification level is much higher than that in parenchyma cells, such as pith cells [33]. In this study, phenylpropanoid biosynthesis-related DEGs, including *GpPAL*-*a*–*GpPAL*-g, *GpC4H*-*a*–*GpC4H*-*b*, *GpCOMT*-*a*–*GpCOMT*-*b*, *GpCCR*-*a*–*GpCCR*-*d*, *GpCAD*-*a*–*GpCAD*-*d*, *GpF5H*-*a*–*GpF5H*-*b*, and *GpPx1*–*GpPx31* genes, were significantly upregulated (Fig. 3). These genes are required for lignin synthesis. In transgenic tobacco, downregulation of *PAL* or *C4H* significantly reduced lignin content [34]. In two *Vicia sativa* varieties, upregulation of *COMT*, *CCR*, and *CAD* led to increased lignin deposition in the cell walls [35]. Overexpression of *F5H* increased lignin content in transgenic *Arabidopsis*, tobacco, and poplar [36]. Transgenic tobacco with 10-fold higher Px activity than the wild type exhibited lignin enrichment in the leaves [37]. Given that the enlargement of cells during aerial stem-to-rhizome transition of *G. pentaphyllum* (Additional file 1: Figure S1), we speculate that the phenylpropanoid biosynthesis-related DEGs are involved in cell-wall expansion to accommodate the enlargement of various cells, in particular vessel and fiber cells, by regulating lignin biosynthesis.

The photoperiod regulates tuber and rhizome formation [9, 38]. The photoperiod-related genes *GpPHYA*, *GpCOL-a*–*GpCOL-g*, *GpCDF*, and *GpFKF1* were identified as DEGs during aerial stem-to-rhizome transition (Table 3). *PHYA* overexpression increased tuber production in short-day potato [39], which is in line with the upregulation of *PHYA* in this study. *CO* family members are involved in tuberization controlled by day length in potato [40]. In lotus, *COL* members control rhizome development [9]. Based on our findings, we speculate that the *COL* homologs detected in *G*. *pentaphyllum* regulate rhizome formation. *CDF* belongs to the large *DOF* transcription factor gene family [41]. In potato, *CDF* overexpression led to early tuber formation [22]. In our study, two *CDF* homologs were downregulated. We speculate that the upregulation of *CDF* expression activates a positive regulator of tuberization to promote tuber formation in potato, whereas the downregulation of *GpCDF* expression represses a negative regulator for rhizome formation to promote aerial stem-to-rhizome transition in *G. pentaphyllum*. FKF1, a clock-controlled protein, degrades CDF by ubiquitin-mediated regulation to control photoperiodic flowering in *Arabidopsis* [42]. The upregulation of *GpFKF1* and downregulation of *GpCDF* observed in our study corroborates an interaction between them and suggests that *GpFKF1* might regulate aerial stem-to-rhizome transition in *G*. *pentaphyllum* by degrading CDF.

In this study, most of the hormone-related genes were assigned to the IAA signaling pathway, and most of them were upregulated during aerial stem-to-rhizome transition (Fig. 4). In *Arabidopsis*, cell-wall acidification-triggered root cell expansion is preceded by increase in IAA signaling [43]. Given the enlargement of cells during stem-to-rhizome transition (Additional file 1: Figure S1), we suggest that IAA-related genes are involved in rhizome formation in *G*. *pentaphyllum*, probably by promoting cell expansion. ABA, CTK, JA, ETH, and BR reportedly also regulate the formation of plant storage organs [21, 44]. Most genes associated with these five hormones were upregulated in this study, indicating that they also regulate the rhizome formation. GA reportedly inhibits storage organ formation [45, 46]. In transgenic potato, overexpression of the GA-biosynthetic gene *GA20ox1* delayed tuberization [46]. In this study, *GpGA20ox* expression was downregulated, whereas the expression of the GA-catabolic gene *GA2ox* was upregulated during stem-to-rhizome transition, suggesting that GA levels are reduced during the transition, thereby promoting the rhizome formation in *G. pentaphyllum*.

Carbohydrate metabolism plays an essential role in plant growth and development as its products are used not only as an energy source, but also for constructing structural cellular components [47]. Starch/sucrose biosynthesis is strongly correlated with the swelling of storage organs [23]. *SUS* and *GBSS* encode key enzymes in starch/sucrose synthesis [48, 49]. The upregulated *SUS* and *GBSS* was in parallel with rhizome enlargement in lotus [9]. In this study, *GpSUS* and *GpGBSS* were significantly upregulated (Table 4), in line with the increased starch accumulation in rhizome cells (Additional file 2: Figure S2). Thus, these two enzymes might promote rhizome formation by providing energy for cell expansion in *G. pentaphyllum*. Starch is also related to gravitropism. In plants, gravity is perceived by specific starch-containing cells located in the root columella and in the starch sheath of stem endodermis [50]. In this study, starch accumulation was observed in the starch sheath of rhizome (Additional file 2: Figure S2), suggesting that the *GpSUS* and *GpGBSS* might indirectly regulate the gravitropic response during stem-to-rhizome transition. SNF1 kinase is a heterotrimer composed of catalytic alpha and regulatory beta and gamma subunits [51] that regulates carbohydrate metabolism [23]. A gene encoding KING1 was significantly upregulated during stem-to-rhizome transition (Table 4), suggesting it is involved in rhizome formation in *G. pentaphyllum*.

### miRNAs and their targets involved in the aerial stem-to-rhizome transition in *G. pentaphyllum*

#### GpmiR166b-*GpECH2* and GpmiR166e-*GpSGT*-*like* modules regulate the aerial stem-to-rhizome transition

miR166 family members modulate various developmental processes by negatively mediating their targets [52]. miR166g and its *HD-ZIP* targets determine the fate of shoot apical meristem and lateral organ formation in *Arabidopsis* [53]. Overexpression of miR166a reduced lateral root by targeting several *HD-ZIP* genes in *Medicago truncatula* [17]. In *Arabidopsis*, miR166 is also involved in the embryogenic transition of somatic cells by regulating its target *PHABULOSA* and the *LEC2*-mediated auxin-related pathway [18]. However, it is unknown whether miR166 family members are involved in rhizome formation. In this study, GpmiR166b and GpmiR166e were significantly downregulated (Table 6 and Fig. 5), indicating that they are involved in aerial stem-to-rhizome transition in *G. pentaphyllum.* This is the first report on the potential regulatory functions of miR166 family members in rhizome formation. GpmiR166b was predicted to target *GpECH2*, which was significantly upregulated (Fig. 5). In *Arabidopsis*, ECH2 promotes cell enlargement during post-mitotic cell expansion in cotyledon development [54]. ECH2 is a peroxisomal enzyme that is essential for IBA-to-IAA conversion through β-oxidation of IBA [54, 55]. The long-standing acid growth theory postulates that IAA triggers cell-wall acidification, thus activating cell wall-loosening enzymes, which enable cell expansion in shoots [56]. A reduction in the IAA level or signaling abolished both cell wall acidification and cellular expansion in *Arabidopsis* roots, supporting the acid growth theory [43]. In this study, *GpECH2* and IAA signaling-related genes were significantly upregulated during stem-to-rhizome transition (Figs. 4 and 5), suggesting that GpmiR166b and its target *GpECH2* promote cell expansion probably by the IBA-to-IAA conversion in the rhizome formation of *G. pentaphyllum*. IBA β-oxidation also leads to the production of acetyl-CoA [55], which can be converted to glucose via the glyoxylate cycle or gluconeogenesis [57] and further polymerized to cellulose, a major structural cell-wall component [24]. The cellulose synthesis-related genes *GpSUS* and *GpCESA* were significantly upregulated in this study (Table 4). Thus, it is possible that GpmiR166b-*GpECH2* is involved in cell wall remodeling for cell expansion via conversion of IBA into acetyl-CoA during stem-to-rhizome transition. GpmiR166e was predicted to target an *GpSGT*-*like* gene, and they exhibited significantly opposite expression trends (Fig. 5). SGT catalyzes the glucosylation of scopoletin to scopolin [58]. In tobacco, scopolin protects IAA from degradation during seedling development [59]. IAA plays fundamental roles in many aspects of plant growth and development [43], which is supported by the increased expression of *GpYUCCA* and IAA signaling-related genes (Table 2 and Fig. 4). Therefore, the GpmiR166e-*GpSGT*-*like* module might protect IAA from inactivation and thereby promote aerial stem-to-rhizome transition in *G. pentaphyllum*.

#### GpmiR156a-*GpSPL6*/*SPL13A* modules regulate the aerial stem-to-rhizome transition

miR156 family members are master regulators of various plant developmental traits [15]. Overexpression of miR156/miR156a prolonged the juvenile phase and delayed flowering in *Arabidopsis*, rice, maize, tomato, and switchgrass [8, 60–63]. In potato, miR156 overexpression induced the production of aerial tubers and regulated the tuberization [64]. In this study, GpmiR156a was significantly upregulated in the two transition phases (Table 6 and Fig. 5), suggesting that it is involved in stem-to-rhizome transition in *G. pentaphyllum*. The miR156a upstream sequence contains several light-regulated motifs, indicating a putative light-mediated regulation of this miRNA [15]. In photoperiod-responsive potato species, miR156a accumulation induced tuberization under short-day, but not long-day condition [15]. In late autumn, the photoperiod gradually shortens, implying that the light-mediated regulation of GpmiR156a is probably involved in aerial stem-to-rhizome transition in *G. pentaphyllum*. Accumulating evidence indicates that miR156 family members can target numerous *GpSPL* genes [65]. *SPL* genes encode plant-specific transcription factors that contain a conserved SBP domain, through which they can recognize and bind specifically to the promoters of target genes, thus affecting plant growth and development [66, 67]. In this study, GpmiR156a putatively targeted *GpSPL6* and *GpSPL13A*, whose expression was significantly downregulated in parallel with the upregulation of GpmiR156a during stem-to-rhizome transition in *G. pentaphyllum* (Fig. 5). *Arabidopsis* has two *SPL13* copies, *SPL13A* and *SPL13B* [68]. *SPL13* plays a crucial role in vegetative and reproductive plant development. In *Arabidopsis*, expression of *SPL13* with a mutated miR156 site delayed leaf production, whereas a loss-of-function mutant of *SPL13* had increased juvenile and rosette leaf numbers [68, 69]. In *Medicago sativa*, *SPL13* overexpression induced severe growth retardation, whereas *SPL13* silencing increased branching and delayed flowering [70]. These finding indicate that *SPL13* represses vegetative phase transition and promoted reproductive phase transition. SPL6 has a conserved DNA-binding domain similar to that of SPL13 [68] and also functions in developmental phase transition [68, 71]. We suggest that GpmiR156a promotes aerial stem-to-rhizome transition in *G. pentaphyllum* by repressing the expression of *GpSPL13A* and *GpSPL6*, which are negative regulators of vegetative phase transition.

#### GpmiR156a and a novel miRNA co.47071 regulate *GpRAV-like* during aerial stem-to-rhizome transition

In addition to *GpSPL6* and *GpSPL13A*, GpmiR156a also targeted *GpRAV-like* gene, together with a novel miRNA Co.47071 (Fig. 5). All members of the *RAV* subfamily contain both AP2/ERF and B3 DNA-binding domains and belong to the AP2/ERF family. *RAV* genes encode transcriptional regulators with various functions in plant developmental and physiological processes [72]. In tobacco, overexpression of a *Glycine max RAV* gene retarded plant growth and reduced root elongation [73]. In *Arabidopsis* and soybean, *GmRAV1* overexpression induced dwarfism, whereas loss-of-function mutant plants had an opposite phenotype [74]. *GmRAV1* promotes root and shoot regeneration by enhancing the expression of cyclins and cyclin-dependent protein kinases to promote cell division [75]. These findings indicate that *RAV* inhibits plant growth probably by inhibiting cell expansion rather than cell division. Our findings suggest that the downregulation of *GpRAV-like* through co-repression by GpmiR156a and miRNA Co.47071 promotes stem-to-rhizome transition in *G. pentaphyllum*, probably by promoting cell expansion.

## Conclusion

Our integrated transcriptome and miRNA analysis revealed a comprehensive molecular network regulating the transition of aerial stem to rhizome in *G. pentaphyllum*. In total, 5428 DEGs were identified, and DEGs associated with gravitropism, cell wall biosynthesis, photoperiod pathway, hormone signaling, and carbohydrate metabolism might largely contribute to aerial stem-to-rhizome transition in this species. Seven DEMs, including four known and three novel miRNAs, were identified. For six DEMs, we were able to predicts targets, which displayed significantly opposite expression trends. The regulatory modules GpmiR166b-*GpECH2*, GpmiR166e-*GpSGT*-*like*, GpmiR156a-*GpSPL13A*/*GpSPL6*, and GpmiR156a/Co.47071-*GpRAV*-*like* likely play important roles in aerial stem-to-rhizome transition. The qRT-PCR results supported the reliability of sequencing data. These results will help elucidate the molecular mechanisms underlying aerial stem-to-rhizome transition in *G. pentaphyllum* and broaden our understanding of developmental phase transitions in plants.

## Methods

### Plant materials

*G. pentaphyllum* plants grew in the field under normal farming conditions in Jishou, Hunan province, China. As shown in Fig. 1, aboveground moderately swelling stem, underground newly formed rhizome, and aerial stem as a control were collected from *G. pentaphyllum* plants when subapical regions of some aerial stems swelled and then grew down into the soil to form rhizomes in late autumn 2018. Aerial stem, aboveground moderately swelling stem, and underground newly formed rhizome were named stage 1, stage 2, and stage 3 of aerial stem-to-rhizome transition, respectively. Part of the samples were immediately frozen in liquid nitrogen and stored at − 80 °C for transcriptome and small RNA-Seq analysis, and the remaining samples were used to investigate histological traits.

### Histological analysis

To investigate histological traits of the three stages of *G. pentaphyllum* stem-to-rhizome transition, the samples were cut into small pieces of approximately 0.5 cm in length and fixed in formalin:acetic acid:70% ethanol solution (5:5:90, v/v/v). The fixed samples were dehydrated in a graded ethanol series and then embedded in paraffin. Sections of 8 μm thickness were cut using a microtome (Leica RM2245; Leica, Nussloch, Germany). Sections were stained with safranin O-fast green and periodic acid-Schiff reagent, respectively, and then observed under a light microscope (Leica DM6000B).

### RNA extraction

Total RNA was extracted from the samples using the easy-spin Plant RNA Kit (Aidlab Biotech, Beijing, China) following the manufacturer’s instructions. Only RNA samples having an 260/280 ratio of 1.8–2.1, an 260/230 ratio of 2.0–2.5, and an RNA integrity number of 8.0 or higher were used for transcriptome and small RNA-Seq. For each transition stage, three biological replicates were prepared.

### Transcriptome sequencing and analysis

cDNA libraries were prepared from 1 μg RNA per sample using a NEBNext® Ultra™ RNA Library Prep Kit for Illumina® (NEB, USA) according to the manufacturer’s recommendations. Nine transcriptome libraries from the above-mentioned three developmental stages were sequenced on an Illumina HiSeq X Ten platform (Biomarker technologies, Beijing, China), generating 150-bp paired-end reads. To obtain high-quality cleaned reads, reads containing adapter and poly-N as well as low-quality reads were removed. The cleaned reads were *de-novo* assembled into transcripts, which were assembled into unigenes, using Trinity v2.5.1 (run parameters: ‘--seqType fq --bflyHeapSpaceMax 20G --min_contig_length 200 --bflyGCThreads 5 --no_run_butterfly --no_run_quantifygraph) [76]. The completeness of transcriptome assembly was assessed by using BUSCO v.3.0.2 (run parameters: -m tran -c 4 -f [77] . The read count for each gene was obtained by mapping the cleaned reads to the assembled transcriptome using RSEM v1.2.19 with default parameters [78].

For functional annotation, assembled unigenes were queried against public databases including NCBI non-redundant protein database (NR, ftp://ftp.ncbi.nih.gov/blast/db/) [79], Swiss-Prot (http://www.uniprot.org/) [80], Gene Ontology (GO, http://www.geneontology.org/) [81], Clusters of Orthologous Groups (COG, http://www.ncbi.nlm.nih.gov/COG/) [82], euKaryotic Orthologous Groups (KOG, http://www.ncbi.nlm.nih.gov/KOG/) [83], orthologous groups of genes (EggNOG, http://eggnogdb.embl.de/) [84] and Kyoto Encyclopedia of Genes and Genomes (KEGG, http://www.genome.jp/kegg/) [85] using BLAST v2.2.31 with an E value cutoff of 1e-5 [86], and Pfam (http://pfam.xfam.org/) database [87] using HMMER v3.1b2 with an E-value cutoff of 1e-10 [88].

Gene expression levels in each sample were normalized as transcripts per million (TPM): TPM = readcount× 1,000,000/ Mapped Reads [89]. Analysis of DEGs was performed using the Bioconductor [90] DESeq2 package v1.6.3 [91] in R v3.1.1 [92] with default parameters, based on a model following a negative binomial distribution [91]. DEGs between three developmental stages were identified based on criteria of FDR < 0.01 and |log_2_ fold change| ≥1. For functional enrichment analysis of the DEGs, GO and KEGG analyses were carried out.

### Small RNA-Seq and data analysis

Nine small RNA libraries were parallelly prepared from isolated total RNA using a NEBNext® Ultra™ small RNA Sample Library Prep Kit for Illumina® (NEB) according to the manufacturer’s protocol. The libraries were sequenced on an Illumina HiSeq X Ten platform (Biomarker technologies, Beijing, China), generating 50-bp paired-end reads. Low-quality reads, contaminating reads with adapters and poly-A tails, and reads without the insert tag were removed. Then, sequences shorter than 18 bp and longer than 35 bp were removed. Finally, rRNAs, tRNAs, snRNAs, snoRNAs, and other noncoding RNAs and repeats were removed by aligning to Rfam (http://rfam.xfam.org/) [93], Silva (http://www.arb-silva.de/) [94], GtRNAdb (http://lowelab.ucsc.edu/GtRNAdb/) [95] and Repbase (http://www.girinst.org/repbase/) [96] databases. Conserved miRNAs were identified by comparing the cleaned small RNA reads with known miRNAs available in miRBase 21 (http://www.mirbase.org/). The alignment was done using Bowtie alignment tool v1.0.0 (run parameters: -v 0) with no mismatch [97]. The unannotated reads were used for prediction of novel miRNAs using miRDeep2 v2.0.5 (run parameters: -g − 1 -l 250 -b 0) [98]. The prediction is based on the biological characteristics of miRNA precursors which have a landmark hairpin-stem-loop structure. Expression levels of miRNAs in each sample were normalized as transcripts per million. DEMs between the transition stages were identified based on criteria of FDR < 0.01 and |log_2_ fold change| ≥1 by using the Bioconductor [90] DESeq2 package v1.6.3 [91] in R v3.1.1 [92] with default parameters.

### miRNA target identification and functional annotation

miRNA-target pairs were predicted using the TargetFinder software v1.6 (run parameters: -c 3) [99]. To predict potential functions of these targets, they were annotated in the NR [79], Swiss-Prot [80], GO [81], COG [82], KEGG [85], KOG [83] and Pfam [87] databases.

### Quantitative real-time PCR analysis

To quantify and validate the expression levels of DEGs, DEMs, and their targets, qRT-PCR was used. For the DEMs, stem-loop qRT-PCR was performed as described by Varkonyi-Gasic et al. [100], with 18S rRNA as a reference gene. For the DEGs and DEM targets, qRT-PCR was conducted as previously described by Guo et al. [101], with actin as a reference gene. Expression levels were expressed relative to the corresponding levels in stage 1 and were calculated by the 2^−ΔΔCT^ method [102]. The significance was tested by Duncan’s multiple range test at the 5% level. Each sample included in the analysis was based on three biological replicates. All qRT-PCR primers used are listed in Additional file 15: Table S7.

## Supplementary information

**Additional file 1: Figure S1.** Anatomical characteristics at different stages in aerial stem-to-rhizome transition in *Gynostemma pentaphyllum*. (a, d) Aerial stem (stage 1). (b, e) Aboveground moderately swelling stem (stage 2). (c, f) Underground newly formed rhizome (stage 3). E: epidermis; Co: cortex; Pe: perivascular fiber; V: vascular bundle; Pi: pith. Bar = 300 μm (a-c); Bar = 50 μm (d-f).

**Additional file 2: Figure S2.** Starch deposition at different stages in aerial stem-to-rhizome transition of *Gynostemma pentaphyllum*. (a, d, g) Aerial stem (stage 1). (b, e, h) Aboveground moderately swelling stem (stage 2). (c, f, i) Underground newly formed rhizome (stage 3). Red granules in the cells indicated by black arrows are starch grains stained with periodic acid-Schiff reagent. E: epidermis; Co: cortex; Pe: perivascular fiber; Ss: starch sheath; V: vascular bundle; Pi: pith. Bar = 50 μm.

**Additional file 3: Figure S3.** (a) Length distribution of assembled unigenes. (b) unigene N50 by expression level. (c) Principal component analysis of the RNA-Seq data. (d) Numbers of differentially expressed genes (DEGs) from pairwise comparisons among different stages of aerial stem-to-rhizome transition in *Gynostemma pentaphyllum*.

**Additional file 4: Figure S4.** Gene Ontology (GO) functional classification of differentially expressed genes (DEGs) for the aerial stem-to-rhizome transition in *Gynostemma pentaphyllum*. (a, d) All DEGs for stage 1 vs stage 2, stage 2 vs stage 3, respectively. (b, e) Up-regulated DEGs for stage 1 vs stage 2, stage 2 vs stage 3, respectively. (c, f) Down-regulated DEGs for stage 1 vs stage 2, stage 2 vs stage 3, respectively.

**Additional file 5: Figure S5.** Kyoto Encyclopedia of Genes and Genomes (KEGG) functional enrichment of differentially expressed genes (DEGs) for the aerial stem-to-rhizome transition in *Gynostemma pentaphyllum*. (a, d) All DEGs for stage 1 vs stage 2, stage 2 vs stage 3, respectively. (b, e) Up-regulated DEGs for stage 1 vs stage 2, stage 2 vs stage 3, respectively (c, f) Down-regulated DEGs for stage 1 vs stage 2, stage 2 vs stage 3, respectively.

**Additional file 6: Figure S6.** Length distribution of miRNAs.

**Additional file 7: Figure S7.** Validation of selected differentially expressed genes (DEGs), as well as differentially expressed miRNAs (DEMs) and their targets by qRT-PCR. (a) DEGs. (b) DEMs. (c) DEMs and their targets. All data in the figure represents the mean values of three independent experiments ± standard deviation (SD) (*n* = 3). Different letters above the columns indicate significant differences at *P* < 0.05.

**Additional file 8: Figure S8.** (a) Phylogenetic relationship between the deduced amino acid sequences of *GpYUCCAs* and *AtYUCCAs*. (b) Phylogenetic relationship between the deduced amino acid sequences of *GpLAZY1* and *AtLAZYs*. (c) Phylogenetic relationship between the deduced amino acid sequences of *GpCOLs* and *AtCOLs*. (d) Phylogenetic relationship between the deduced amino acid sequences of *GpCDF* and other plant *CDFs*. Notes: *Gp*: *Gynostemma pentaphyllum*; *At*: *Arabidopsis thaliana*; *St*: *Solanum tuberosum*. Black arrows indicate protein associated with photoperiod and gravitropism in *Arabidopsis* or *Solanum tuberosum*, and red arrows indicate putative proteins in *G. pentaphyllum*. Accession numbers: AtYUCCA1, number: NP_194980; AtYUCCA2, number: NP_193062; AtYUCCA3, number: NP_171955; AtYUCCA4, number: NP_196693; AtYUCCA5, number: NP_199202; AtYUCCA6, number: NP_001190399; AtYUCCA7, number: NP_180881; AtYUCCA8, number: NP_194601; AtYUCCA9, number: NP_171914; AtYUCCA10, number: NP_175321; AtYUCCA11, number: NP_173564; AtLAZY1, number: NP_196913; AtLAZY2, number: NP_173183; AtLAZY3, number: NP_001117313; AtLAZY4, number: NP_177393; AtLAZY5, number: NP_189119; AtLAZY6, number: NP_850639; AtCOL1, number: NP_197089; AtCOL2, number: NP_186887; AtCOL3, number: NP_180052; AtCOL4, number: NP_197875; AtCOL5, number: NP_568863; AtCOL7, number: NP_177528; AtCOL9, number: NP_187422; AtCOL12, number: NP_188826; AtCOL16, number: NP_173915; AtCDF1, number: NP_197695; AtCDF2, number: NP_851106; AtCDF3, number: NP_190334; AtCDF4, number: NP_180961; AtCDF5, number: NP_177116; AtCDF6, number: NP_174001; StCDF1, number: NP_001305611.

**Additional file 9: Table S1.** Length distribution of assembled transcripts and unigenes.

**Additional file 10: Table S2.** BUSCO analysis of transcriptome assembly in *Gynostemma pentaphyllum*.

**Additional file 11: Table S3.** Detailed list of differentially expressed genes in during aerial stem-to-rhizome transition *Gynostemma pentaphyllum.*

**Additional file 12: Table S4.** Classification of small RNAs.

**Additional file 13: Table S5.** Detailed information of known and novel miRNAs in *Gynostemma pentaphyllum.*

**Additional file 14: Table S6.** Predicted targets of differentially expressed miRNAs during the aerial stem-to-rhizome transition of *Gynostemma pentaphyllum.*

**Additional file 15: Table S7.** Primers used for quantitative RT-PCR in the study.

## Data Availability

The Illumina sequencing reads have been deposited in the National Center for Biotechnology Information (NCBI) Sequence Read Archive under the accession number SRP197408, which is associated with BioProject number PRJNA541825 (https://www.ncbi.nlm.nih.gov/bioproject/PRJNA541825). The Transcriptome Shotgun Assembly project has been deposited at GenBank under the accession GHVI00000000, and the version described in this paper is the first version (GHVI01000000).

## Abbreviations

ABA: Abscisic acid
AP2: APETALA2
ARP 2/3 complex: Actin-related protein 2/3 complex
BR: Brassinosteroid
BUSCO: Bench-marking universal single-copy orthologs
C4H: Trans-cinnamate 4-monooxygenase
CAD: Cinnamyl-alcohol dehydrogenase
CCR: Cinnamoyl-CoA reductase
CDF: Cyclic dof factor
CESA: Cellulose synthase
COL: CONSTANS-like
COMT: Caffeic acid 3-O-methyltransferase
CTK: Cytokinin
DEGs: Differentially expressed genes
DEMs: Differentially expressed miRNAs
ECH2: Enoyl-CoA hydratase 2
ETH: Ethylene
F5H: Ferulate-5-hydroxylase
FKF1: Flavin-binding kelch repeat F-box protein 1
GA: Gibberellin acid
GBSS: Granule-bound starch synthase
GO: Gene Ontology
HD-ZIP: Homeodomain-leucine zipper
IAA: Auxin
JA: Jasmonic acid
KEGG: Kyoto Encyclopedia of Genes and Genomes
KING1: SNF1-related protein kinase regulatory subunit gamma-1
PAL: Phenylalanine ammonia-lyase
PCA: Principal component analysis
PHYA: Phytochrome A
Px: peroxidase
qRT-PCR: Quantitative real-time PCR
RAV: Related to ABI3/VP1
RNA-Seq: RNA-Sequencing
rRNA: Ribosomal RNA
SA: Salicylic acid
SGT: Scopoletin glucosyltransferase
snoRNA: Small nucleolar RNA
SPL: SQUAMOSA PROMOTER BINDING PROTEIN-LIKE
SUS: Sucrose synthase
tRNA: Transfer RNA
YUCCA: Indole-3-pyruvate monooxygenase
