# Supplementary figures and images for: Integrated transcriptome and miRNA analysis uncovers molecular regulators of aerial stem-to-rhizome transition in the medical herb Gynostemma pentaphyllum
Source: BMC Genomics. 2019 Nov 15;20:865. doi: 10.1186/s12864-019-6250-8 (PMC6858658; doi:10.1186/s12864-019-6250-8)

**a**

Co E  
V  
Pe  
Pi

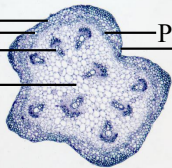**b**

Co  
V  
E  
Pe  
Pi

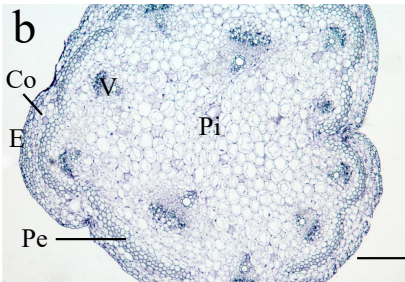**c**

E  
Co  
Pe  
V  
Pi

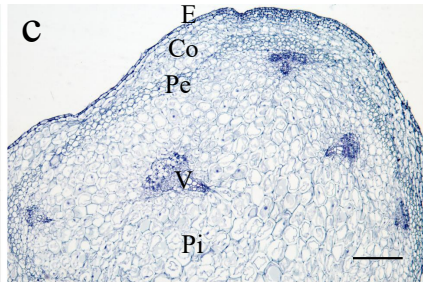**d**

Co  
Pe

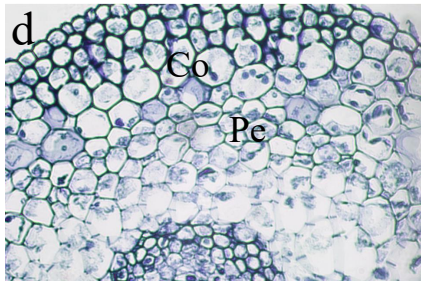**e**

Co  
Pe

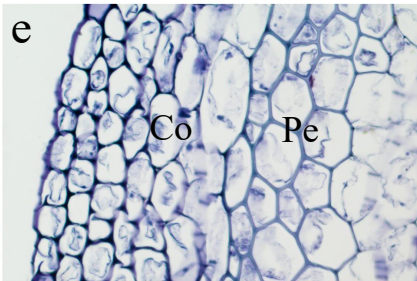**f**

Co  
Pe

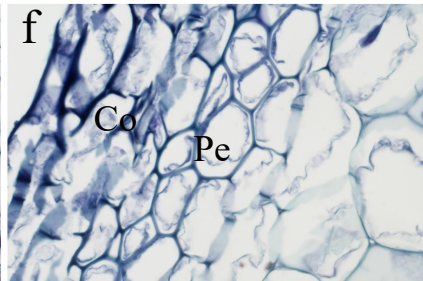

Supplement: Supplementary file 1 — Additional file 1: Figure S1. Anatomical characteristics at different stages in aerial stem-to-rhizome transition in Gynostemma pentaphyllum. (a, d) Aerial stem (stage 1). (b, e) Aboveground moderately swelling stem (stage 2). (c, f) Underground newly formed rhizome (stage 3). E: epidermis; Co: cortex; Pe: perivascular fiber; V: vascular bundle; Pi: pith. Bar = 300 μm (a-c); Bar = 50 μm (d-f). [file 12864_2019_6250_MOESM1_ESM.pdf]

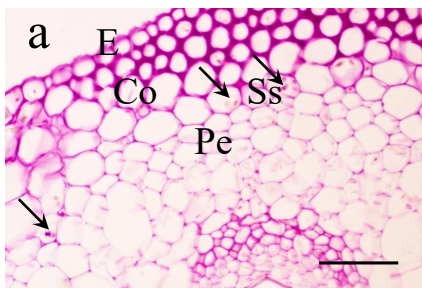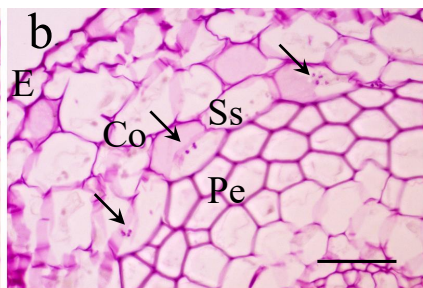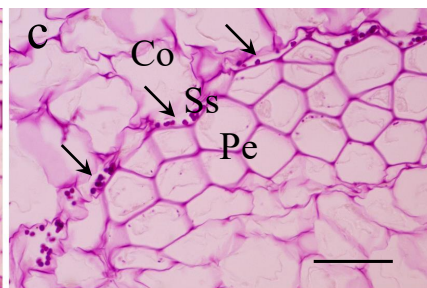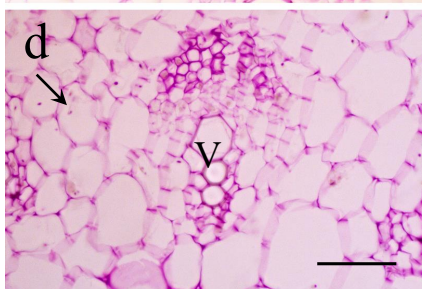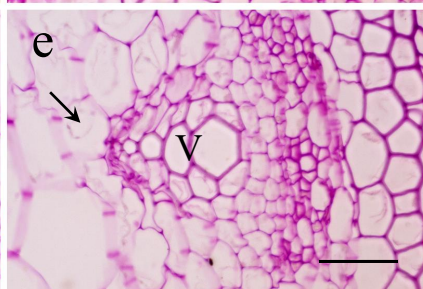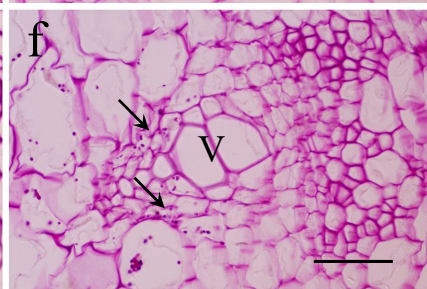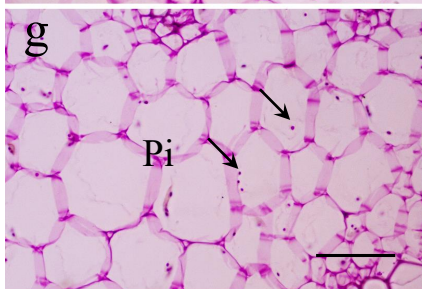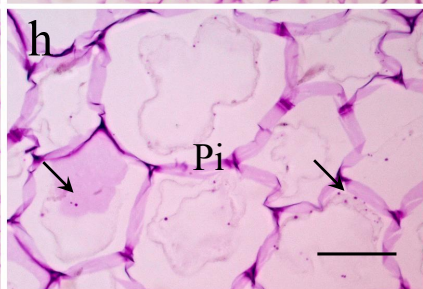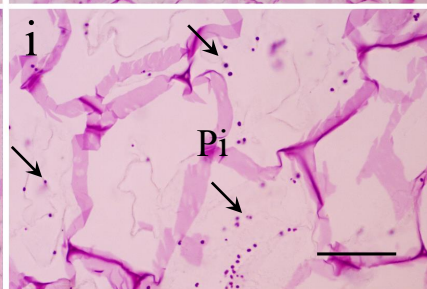

Supplement: Supplementary file 2 — Additional file 2: Figure S2. Starch deposition at different stages in aerial stem-to-rhizome transition of Gynostemma pentaphyllum. (a, d, g) Aerial stem (stage 1). (b, e, h) Aboveground moderately swelling stem (stage 2). (c, f, i) Underground newly formed rhizome (stage 3). Red granules in the cells indicated by black arrows are starch grains stained with periodic acid-Schiff reagent. E: epidermis; Co: cortex; Pe: perivascular fiber; Ss: starch sheath; V: vascular bundle; Pi: pith. Bar = 50 μm. [file 12864_2019_6250_MOESM2_ESM.pdf]

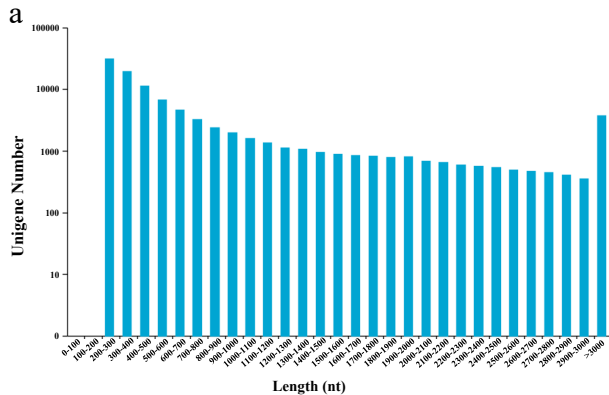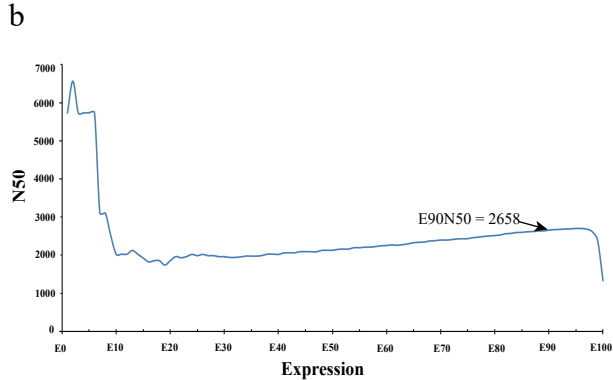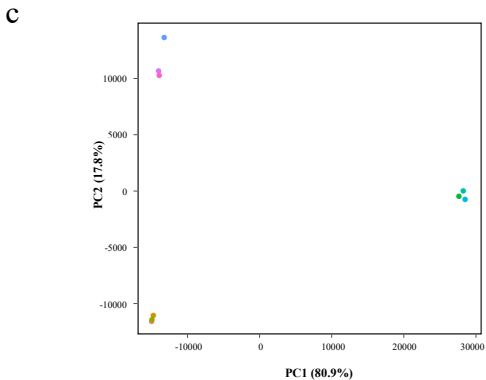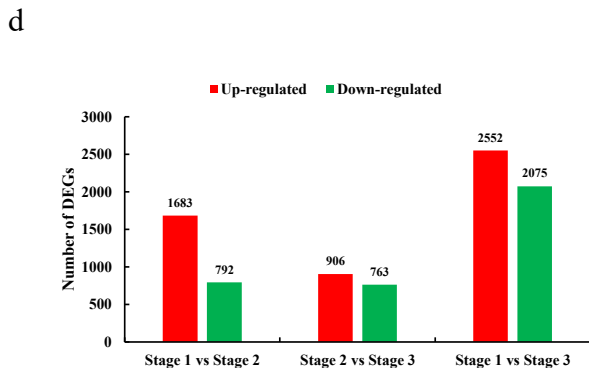

Supplement: Supplementary file 3 — Additional file 3: Figure S3. (a) Length distribution of assembled unigenes. (b) unigene N50 by expression level. (c) Principal component analysis of the RNA-Seq data. (d) Numbers of differentially expressed genes (DEGs) from pairwise comparisons among different stages of aerial stem-to-rhizome transition in Gynostemma pentaphyllum. [file 12864_2019_6250_MOESM3_ESM.pdf]

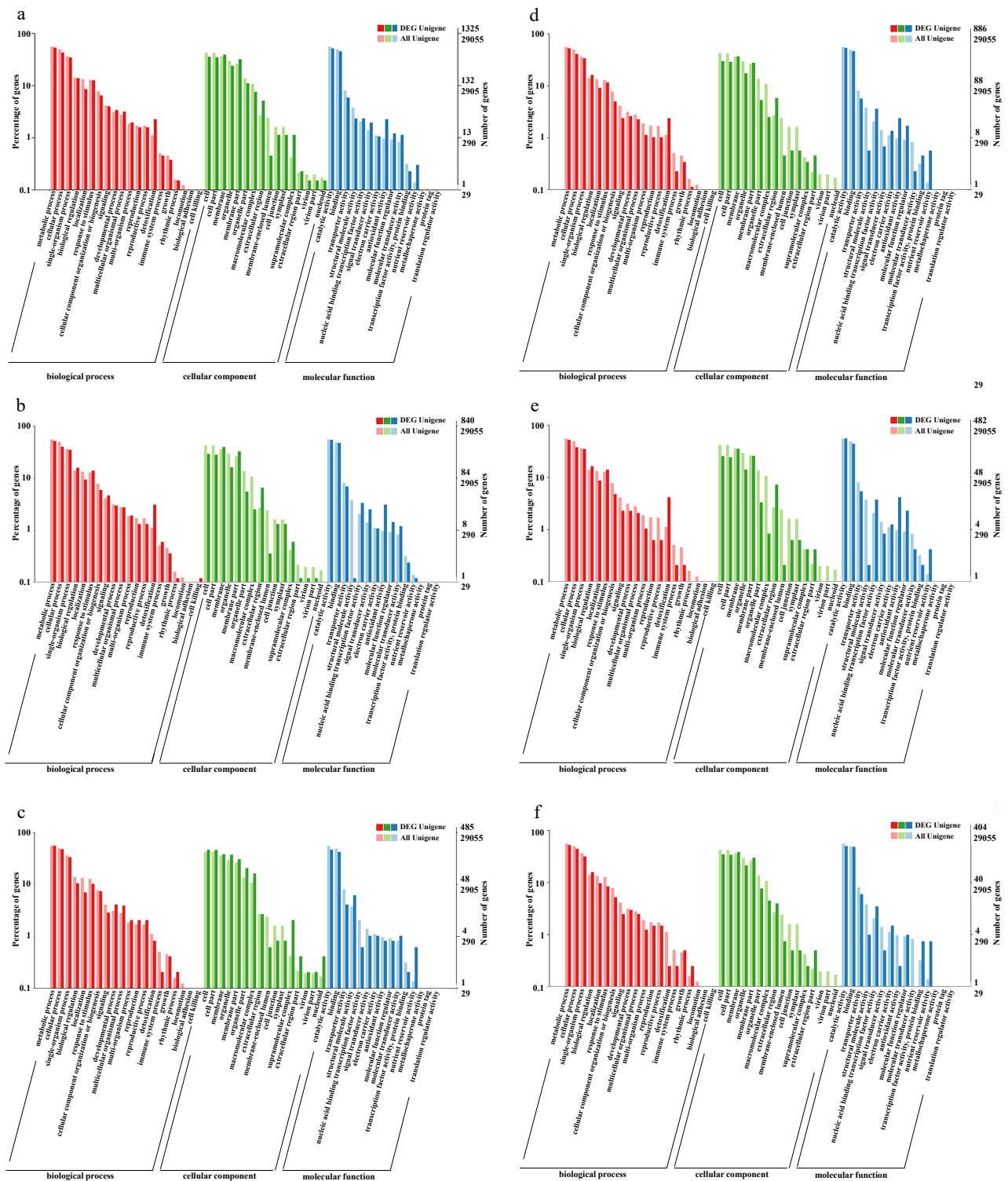

Supplement: Supplementary file 4 — Additional file 4: Figure S4. Gene Ontology (GO) functional classification of differentially expressed genes (DEGs) for the aerial stem-to-rhizome transition in Gynostemma pentaphyllum. (a, d) All DEGs for stage 1 vs stage 2, stage 2 vs stage 3, respectively. (b, e) Up-regulated DEGs for stage 1 vs stage 2, stage 2 vs stage 3, respectively. (c, f) Down-regulated DEGs for stage 1 vs stage 2, stage 2 vs stage 3, respectively. [file 12864_2019_6250_MOESM4_ESM.pdf]

a

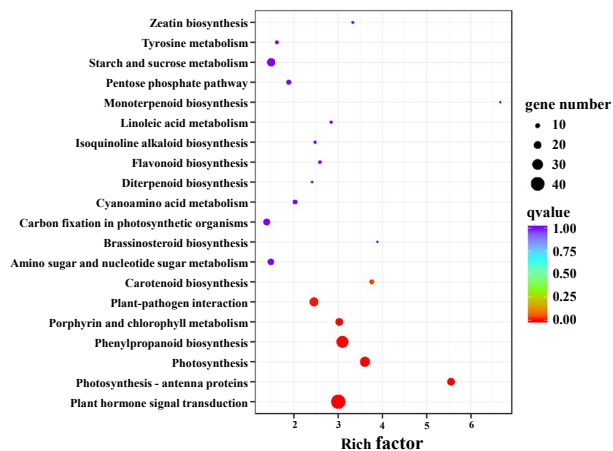

d

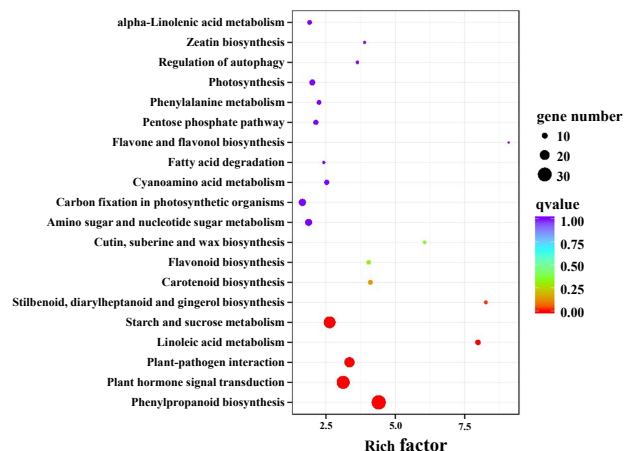

b

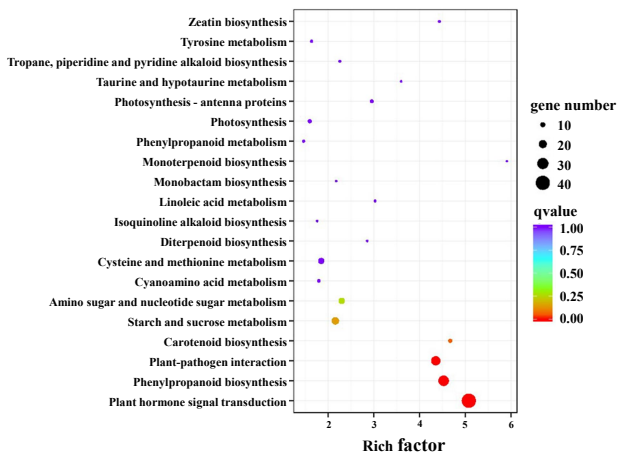

e

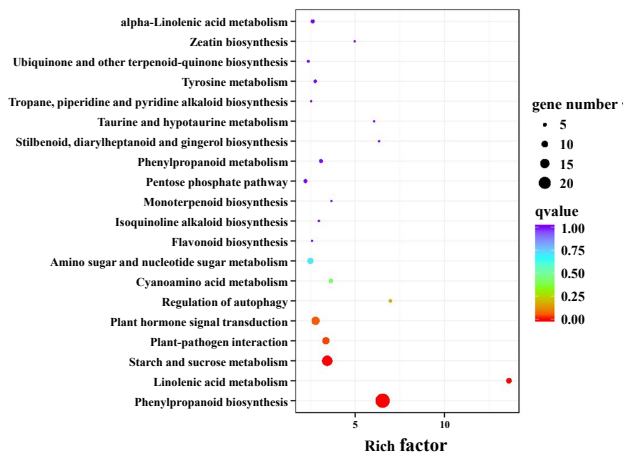

f

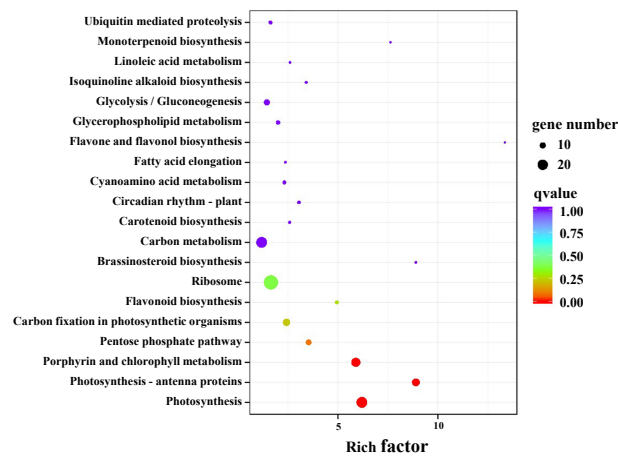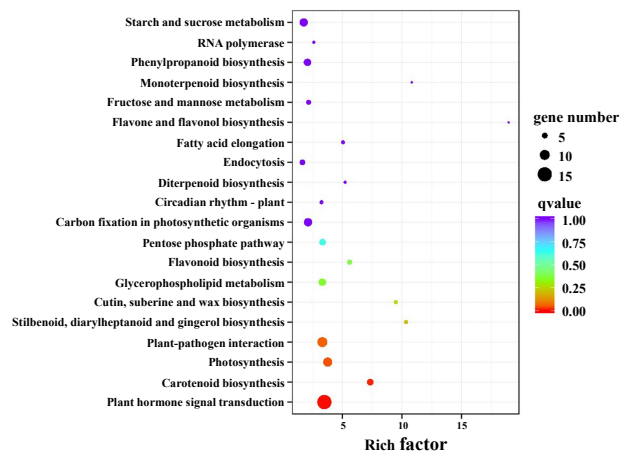

Supplement: Supplementary file 5 — Additional file 5: Figure S5. Kyoto Encyclopedia of Genes and Genomes (KEGG) functional enrichment of differentially expressed genes (DEGs) for the aerial stem-to-rhizome transition in Gynostemma pentaphyllum. (a, d) All DEGs for stage 1 vs stage 2, stage 2 vs stage 3, respectively. (b, e) Up-regulated DEGs for stage 1 vs stage 2, stage 2 vs stage 3, respectively (c, f) Down-regulated DEGs for stage 1 vs stage 2, stage 2 vs stage 3, respectively. [file 12864_2019_6250_MOESM5_ESM.pdf]

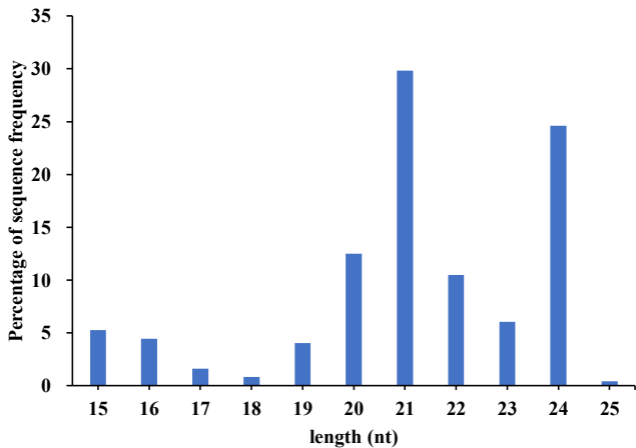

Supplement: Supplementary file 6 — Additional file 6: Figure S6. Length distribution of miRNAs. [file 12864_2019_6250_MOESM6_ESM.pdf]

a

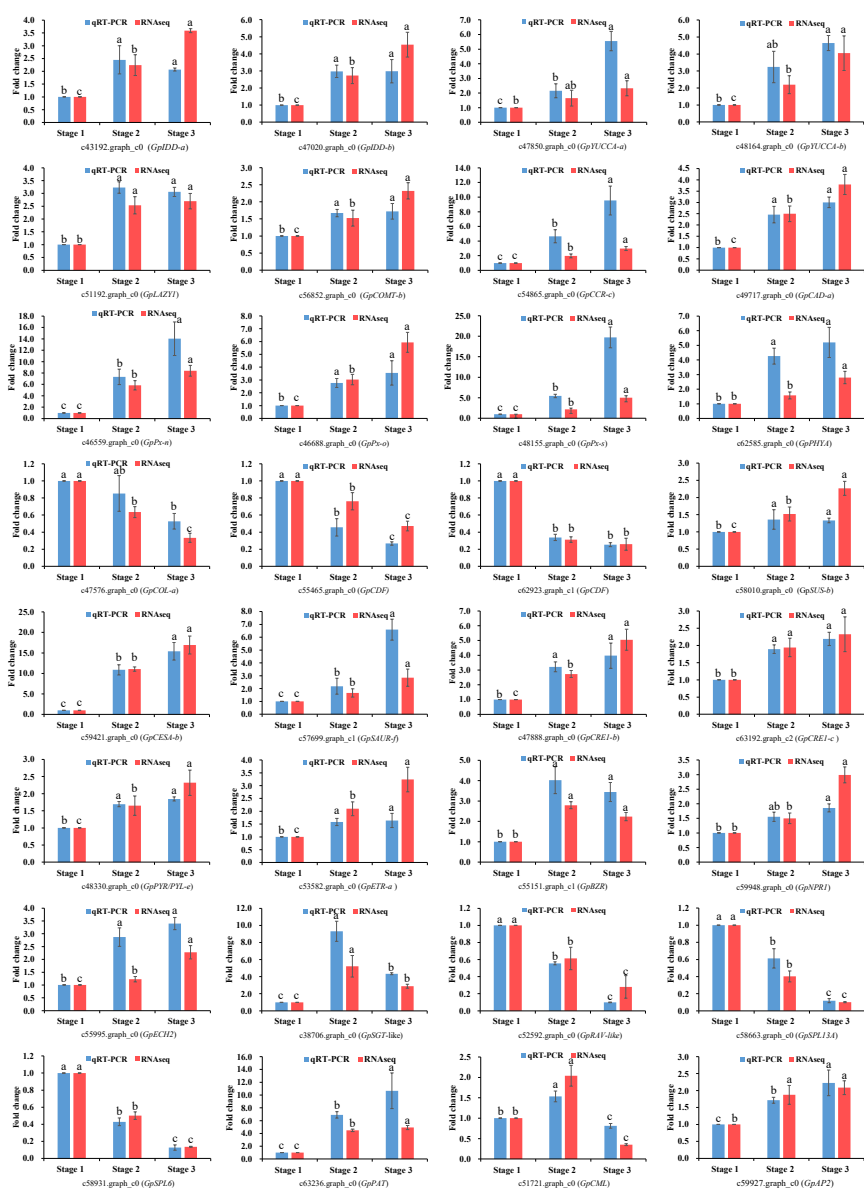

b

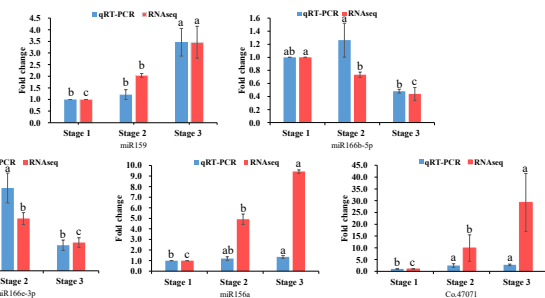

c

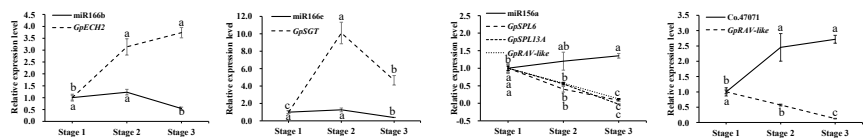

Supplement: Supplementary file 7 — Additional file 7: Figure S7. Validation of selected differentially expressed genes (DEGs), as well as differentially expressed miRNAs (DEMs) and their targets by qRT-PCR. (a) DEGs. (b) DEMs. (c) DEMs and their targets. All data in the figure represents the mean values of three independent experiments ± standard deviation (SD) (n = 3). Different letters above the columns indicate significant differences at P < 0.05. [file 12864_2019_6250_MOESM7_ESM.pdf]

**a**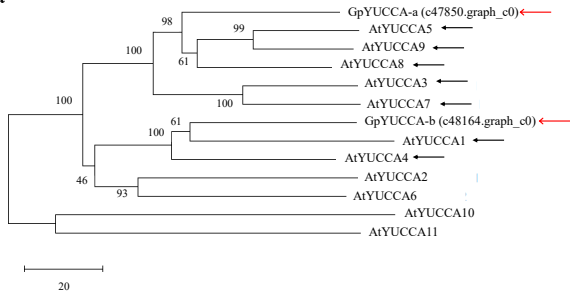**b**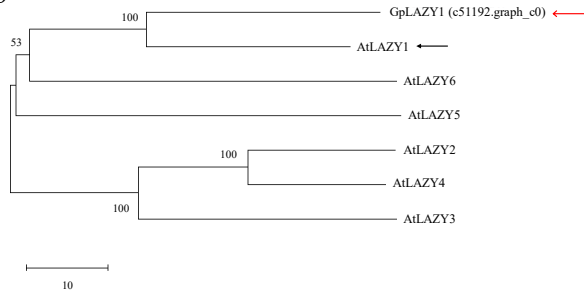**c**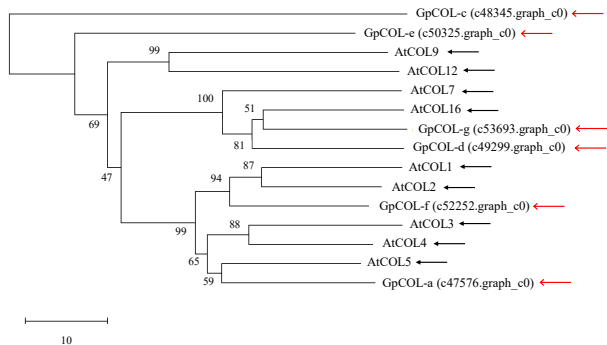**d**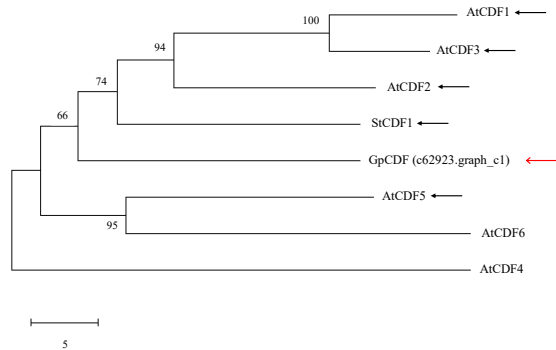

Supplement: Supplementary file 8 — Additional file 8: Figure S8. (a) Phylogenetic relationship between the deduced amino acid sequences of GpYUCCAs and AtYUCCAs. (b) Phylogenetic relationship between the deduced amino acid sequences of GpLAZY1 and AtLAZYs. (c) Phylogenetic relationship between the deduced amino acid sequences of GpCOLs and AtCOLs. (d) Phylogenetic relationship between the deduced amino acid sequences of GpCDF and other plant CDFs. Notes: Gp: Gynostemma pentaphyllum; At: Arabidopsis thaliana; St: Solanum tuberosum. Black arrows indicate protein associated with photoperiod and gravitropism in Arabidopsis or Solanum tuberosum, and red arrows indicate putative proteins in G. pentaphyllum. Accession numbers: AtYUCCA1, number: NP_194980; AtYUCCA2, number: NP_193062; AtYUCCA3, number: NP_171955; AtYUCCA4, number: NP_196693; AtYUCCA5, number: NP_199202; AtYUCCA6, number: NP_001190399; AtYUCCA7, number: NP_180881; AtYUCCA8, number: NP_194601; AtYUCCA9, number: NP_171914; AtYUCCA10, number: NP_175321; AtYUCCA11, number: NP_173564; AtLAZY1, number: NP_196913; AtLAZY2, number: NP_173183; AtLAZY3, number: NP_001117313; AtLAZY4, number: NP_177393; AtLAZY5, number: NP_189119; AtLAZY6, number: NP_850639; AtCOL1, number: NP_197089; AtCOL2, number: NP_186887; AtCOL3, number: NP_180052; AtCOL4, number: NP_197875; AtCOL5, number: NP_568863; AtCOL7, number: NP_177528; AtCOL9, number: NP_187422; AtCOL12, number: NP_188826; AtCOL16, number: NP_173915; AtCDF1, number: NP_197695; AtCDF2, number: NP_851106; AtCDF3, number: NP_190334; AtCDF4, number: NP_180961; AtCDF5, number: NP_177116; AtCDF6, number: NP_174001; StCDF1, number: NP_001305611. [file 12864_2019_6250_MOESM8_ESM.pdf]
